# Metabolic changes of human induced pluripotent stem cell-derived cardiomyocytes and teratomas after transplantation

**DOI:** 10.1016/j.isci.2024.111234

**Published:** 2024-10-23

**Authors:** Yusuke Soma, Shugo Tohyama, Akiko Kubo, Tomoteru Yamasaki, Noriko Kabasawa, Kotaro Haga, Hidenori Tani, Yuika Morita-Umei, Tomohiko C. Umei, Otoya Sekine, Masashi Nakamura, Taijun Moriwaki, Sho Tanosaki, Shota Someya, Yujiro Kawai, Masatoshi Ohno, Yoshikazu Kishino, Hideaki Kanazawa, Jun Fujita, Ming-Rong Zhang, Makoto Suematsu, Keiichi Fukuda, Masaki Ieda

**Affiliations:** 1 Department of Clinical Regenerative Medicine, Fujita Medical Innovation Center, Fujita Health University, Ota-ku, Tokyo 144-0041, Japan; 2 Department of Cardiology, Keio University School of Medicine, Shinjuku-ku, Tokyo 160-8582, Japan; 3 Department of Biochemistry, Keio University School of Medicine, Shinjuku-ku, Tokyo 160-8582, Japan; 4 Department of Cardiovascular Surgery, Keio University School of Medicine, Shinjuku-ku, Tokyo 160-8582, Japan; 5 WPI-Bio2Q, Keio University School of Medicine, Shinjuku-ku, Tokyo 160-8582, Japan; 6 Center for Prevention Medicine, Keio University School of Medicine, Minato-ku, Tokyo 106-0041, Japan; 7 Department of Advanced Nuclear Medicine Sciences, Institute for Quantum Medical Science, National Institutes for Quantum Science and Technology, Inage-ku, Chiba 263-8555, Japan; 8 Heartseed Inc, Minato-ku, Tokyo 105-0023, Japan; 9 Kanagawa Institute of Industrial Science and Technology (KISTEC), Kawasaki-ku, Kawasaki 210-0821, Japan; 10 Department of Pathology & Immunology, Baylor College of Medicine, Houston, TX 77030, USA; 11 Central Institute for Experimental Medicine and Life Science, Kawasaki-ku, Kawasaki 210-0821, Japan

**Keywords:** cardiovascular medicine, cell biology

## Abstract

Cardiac regenerative therapy using human induced pluripotent stem cell-derived cardiomyocytes (hiPSC-CMs) has been applied in clinical settings. Herein, we aimed to investigate the *in vivo* metabolic profiles of hiPSC-CM grafts. RNA sequencing and imaging mass spectrometry were performed in the present study, which revealed that hiPSC-CM grafts matured metabolically over time after transplantation. Glycolysis, which was active in the hiPSC-CM grafts immediately after transplantation, shifted to fatty acid oxidation. Additionally, we examined the metabolic profile of teratomas that may form when non-CMs, including undifferentiated human induced pluripotent stem cells (hiPSCs), remain in transplanted cells. The upregulated gene expression of amino acid transporters and the high accumulation of amino acids, such as methionine and aromatic amino acids, were observed in the teratomas. We show that subcutaneous teratomas derived from undifferentiated hiPSCs can be detected *in vivo* using positron emission tomography with [^18^F]fluorophenylalanine ([^18^F]fPhe). These results provided insights into the clinical application of cardiac regenerative therapy.

## Highlights

• hiPSC-CMs become morphologically and functionally mature after transplantation
• hiPSC-CM grafts undergo metabolic maturation from glycolysis to fatty acid oxidation
• LAT1 is highly expressed and amino acids accumulate in hiPSC-derived teratomas
• [^18^F]fPhe detects hiPSC-derived teratomas in skeletal muscle and subcutaneous tissue

## Graphical abstract

**Figure:**
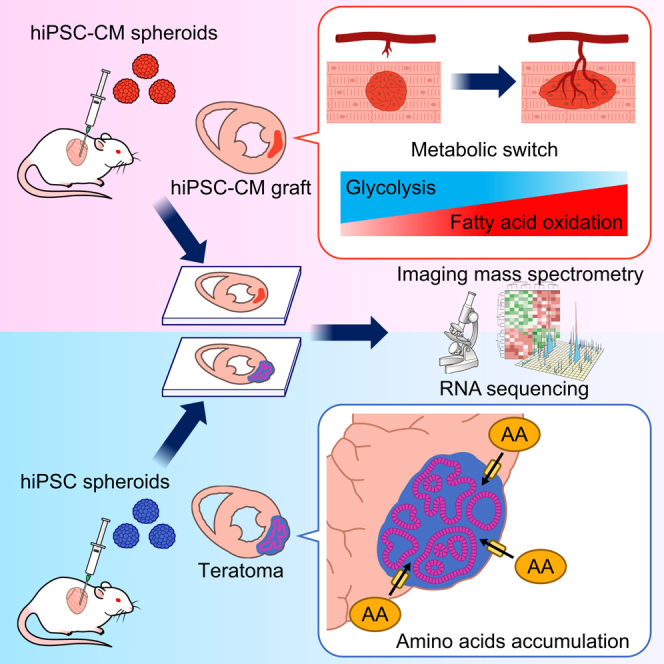

## Introduction

The number of patients with heart failure (HF) is increasing in the current aging society.^1^ Regenerative therapy using human pluripotent stem cells (hPSCs), such as human embryonic stem cells^2^ and human induced pluripotent stem cells (hiPSCs)^3^^,^^4^ can be an alternative to heart transplantation in patients with severe HF. Our team developed metabolism-based cardiomyocyte (CM) production systems^5^^,^^6^^,^^7^^,^^8^^,^^9^^,^^10^^,^^11^^,^^12^ and has initiated a clinical trial, titled the LAPiS study, where hiPSC-derived cardiac spheroids (hiPSC-CSs) have been directly transplanted into the cardiac muscle layer for treating patients with severe ischemic cardiomyopathy.^13^

hPSCs differentiate into CMs; however, the resulting hPSC-derived CMs (hPSC-CMs) are as immature as fetal CMs.^14^ Long-term culture in a two-dimensional (2D) culture system morphologically matures hiPSC-CMs only up to the neonatal stage.^15^ To date, several studies have focused on hiPSC-CM maturation in 2D culture using appropriate substrates^16^ and extracellular matrix,^17^^,^^18^ co-culture with non-CMs,^19^ supplementation of some hormones,^20^^,^^21^^,^^22^ and through metabolic pathway modulations.^23^^,^^24^^,^^25^^,^^26^ hPSC-CMs cultured in a three-dimensional (3D) culture system become morphologically and functionally more mature; however, this maturation is not equivalent but close to adult CM maturation.^27^^,^^28^^,^^29^ Additionally, hPSC-CMs metabolically mature in a 3D culture system.^30^^,^^31^ Fetal CMs depend on glycolysis, but undergo oxidative phosphorylation and mainly use fatty acids as an energy source in the late neonatal and subsequent phases.^32^^,^^33^ Similarly, hPSC-CMs initially depend on glycolysis. By culturing hPSC-CMs in 3D formats, such as spheroids or engineered heart tissues, fatty acid oxidation (FAO) becomes superior to glycolysis.^30^^,^^31^ Furthermore, hPSC-CMs transplanted into the host myocardium mature morphologically *in vivo*.^12^^*,*^^14^^,^^15^^,^^34^^,^^35^^,^^36^^,^^37^^,^^38^^,^^39^ For example, we transplanted hiPSC-CSs into the myocardium of cynomolgus monkeys with myocardial infarction and showed large engraftment, morphological maturation of transplanted hiPSC-CMs, improvement in cardiac function 3 months after transplantation, and extremely low occurrence of arrhythmia.^12^ However, the degree of how mature hPSC-CMs become metabolically *in vivo* is unknown.

If non-CMs, including undifferentiated hPSCs, remain present in transplanted cells, they can induce tumors, such as teratomas, at the transplantation site or ectopically. hPSC-derived teratomas (hPSC-Ts) were reportedly formed when 1.0 × 10^6^ fibroblasts containing only 0.025% hPSCs were injected into the legs of immunodeficient mice.^40^ Also, teratomas were formed when cells in the process of differentiating into CMs from mouse PSCs via embryoid body formation were transplanted into the hearts of mice.^41^ Moreover, hPSC-Ts developed after pancreatic islet beta cells, induced to differentiate from autologous hPSCs, were transplanted into the deltoid muscle of a patient.^42^ The persisting issues include elucidation of the metabolism of hPSC-Ts and establishment of noninvasive techniques for hPSC-T imaging. An animal experiment using rats indicated that hPSC-Ts larger than 8 mm^3^ could be detected using magnetic resonance imaging (MRI).^43^ However, as the strength of the MRI magnetic field for small animals is often higher than that in clinical settings,^43^ it is possible that hPSC-Ts in humans cannot be detected until they grow larger. Although [^18^F]fluorodeoxyglucose-PET ([^18^F]FDG-PET) is useful in detecting malignant tumors, detection of hPSC-Ts using [^18^F]FDG-PET remains challenging owing to the benign nature of the cells.^44^ PET/SPECT probes that connect with integrins can detect hPSC-Ts^44^^,^^45^; however, they have not yet been widely used in clinical practice. Moreover, PET can detect hPSC-Ts using genetically modified hPSCs; nevertheless, the safety of this method remains a concern.^46^^,^^47^ The development of technology for detecting hPSC-Ts is necessary for promoting the application of regenerative therapy using hPSC-CMs and other hPSC-derived derivatives.

In this study, we aimed to investigate the metabolic profiles of hiPSC-CM and hiPSC-T grafts. We transplanted hiPSC-CSs or undifferentiated hiPSC spheroids into the hearts of immunodeficient mice. We then trimmed and collected hiPSC-CM or -T grafts via laser capture microdissection (LCM), and compared their metabolic profiles by performing RNA sequencing of the grafts. Additionally, we performed imaging mass spectrometry (IMS) to evaluate the metabolism of the hiPSC-CM or -T grafts. Based on the obtained metabolic characteristics, we performed PET imaging to detect hiPSC-Ts.

## Results

### hiPSC-CM grafts matured morphologically and functionally *in vivo*

**Figure 1:**
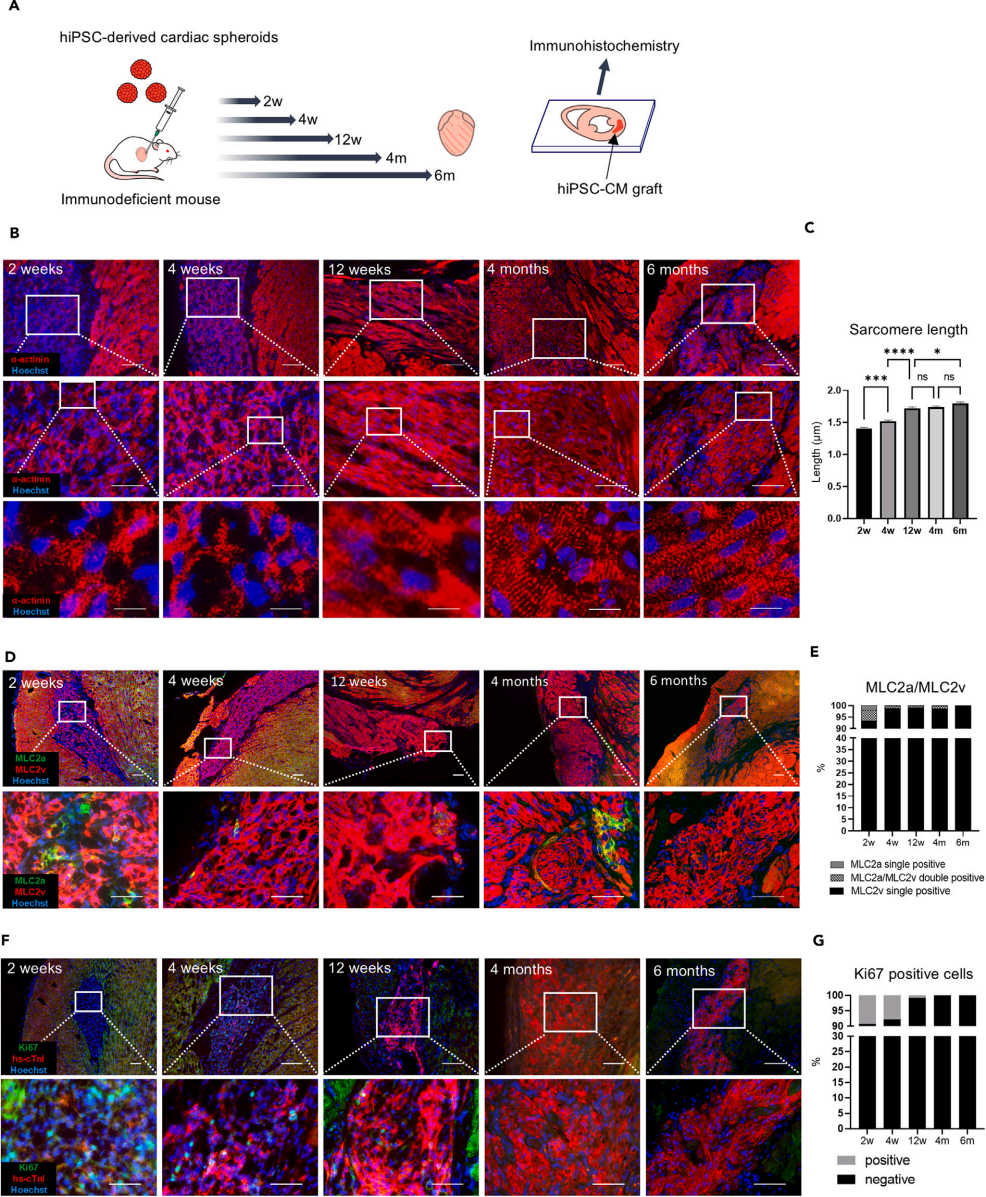
Maturation of hiPSC-CMs transplanted into the myocardium of NOG mice (A) Scheme of the experiment. (B) Graft staining for sarcomere structure (α-actinin, red). Scale bar, (upper) 100 μm; (middle) 50 μm; and (lower) 10 μm. (C) Quantification of sarcomere length (*n* = 20 sarcomeres at each time point. Tukey’s multiple comparisons test. ∗*p* < 0.05, ∗∗∗*p* < 0.0005, ∗∗∗∗*p* < 0.0001. Error bars represent SEM). (D) Graft staining for the mature ventricular phenotype (MLC2v, red) or the immature ventricular/atrial/nodal cardiomyocyte phenotype (MLC2a, green). (E) The proportion of MLC2a- and MLC2v-positive CMs. (F) Graft staining for Ki67 (green) and human-specific cTnI (red). hs: human-specific. (G) The proportion of Ki67-positive cells in the graft.

hiPSCs were induced to differentiate into CMs, and only hiPSC-CMs were purified using glucose- and glutamine (Gln)-free medium supplemented with lactate.^5^^,^^6^^,^^7^ hiPSC-CSs were obtained by culturing hiPSC-CMs in microwell plates for a week.^48^ These hiPSC-CSs comprised approximately 1,000 hiPSC-CMs. Then, these hiPSC-CSs were transplanted into the myocardium of non-obese diabetic (NOD)/Shi-scid IL-2RγKO (NOG) mice. The maturation of these cells was evaluated via immunohistochemistry at 2 weeks, 4 weeks, 12 weeks, 4 months, and 6 months post-transplantation (Figure 1A). The hiPSC-CM grafts were identified by staining with human-specific cardiac troponin I (cTnI) (Figure 1F). Moreover, the grafts were also identified by staining human nuclei at 2 and 4 weeks because they were immature and showed low expression of cTnI at these time points (Figure S1A). Consistent with previous reports,^12^^,^^14^^,^^15^^,^^34^^,^^35^^,^^36^^,^^37^^,^^38^^,^^39^ the transplanted hiPSC-CMs became larger and more aligned (Figure 1B). Sarcomere structures gradually became more distinct. Furthermore, the sarcomere length increased from 1.405 ± 0.019 μm at 2 weeks to 1.800 ± 0.018 μm at 6 months (Figure 1C). Mature ventricular CMs, MLC2v-positive and MLC2a-negative cells, were present in the graft, and the proportion of these cells in the graft was 93.3% at 2 weeks. The proportion was over 98% at 4 weeks and reached 100% at 6 months (Figure 1D and 1E). The isoform of TnI switched from slow skeletal TnI to cTnI during maturation. In hiPSC-CM grafts, the number of cTnI-positive cells increased over time (Figure 1F). The proportion of Ki67-positive cells in the graft was approximately 9.36% at 2 weeks, and there were no Ki67-positive cells in the graft at 4 and 6 months, indicating that the transplanted hiPSC-CMs matured during this period (Figure 1F–1G). The engrafted hiPSC-CM tissues contained a high density of host-derived microvessels, and the number of these vessels increased in the period from 2 weeks to 6 months (Figure S1A and S1B). Abundant host-derived microvessels probably contributed to engraftment.

### hiPSC-CM grafts underwent metabolic maturation from glycolysis to FAO *in vivo*

**Figure 2:**
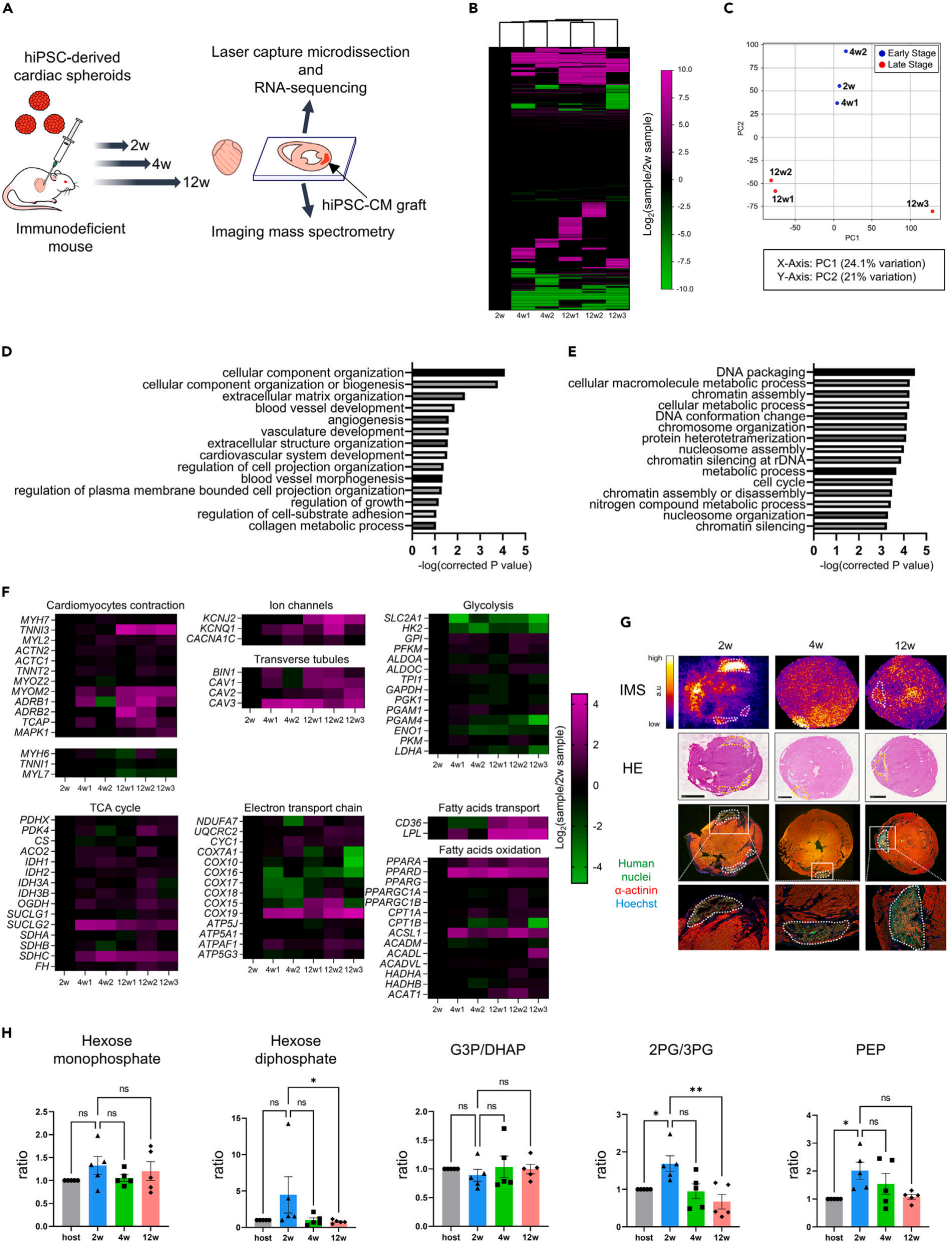
Functional and metabolic maturation of hiPSC-CM grafts evaluated using RNA sequencing and IMS (A) Scheme of the experiment. (B) Heatmap showing RNA sequencing of hiPSC-CM grafts (2 weeks, *n* = 1; 4 weeks, *n* = 2; and 12 weeks, *n* = 3). (C) Principal component analysis of the early-stage group (2 weeks and 4 weeks) and the late-stage group (12 weeks). (D) Gene ontology analysis of the genes that were upregulated in the late-stage group compared with those in the early-stage group. (E) Gene ontology analysis of the genes that were downregulated in the late-stage group compared with those in the early-stage group. (F) Heatmap showing characteristic gene expression of hiPSC-CM grafts. (G) Representative data of the accumulation of hexose diphosphate in hiPSC-CM grafts and host myocardium measured by IMS. Scale bar, 1 mm. (H) The ratio of glycolytic metabolites between hiPSC-CM grafts and host myocardium measured via IMS at 2, 4, and 12 weeks (*n* = 5, respectively. Dunn’s multiple comparisons test. ∗*p* < 0.05. Error bars represent SEM). G3P/DHAP, glyceraldehyde-3-phosphate/dihydroxyacetone phosphate; 2PG/3PG, 2-phosphoglycerate/3-phosphoglycerate; PEP, phosphoenolpyruvic acid.

hiPSC-CSs were transplanted into the hearts of NOG mice, and the mice were dissected at 2, 4, and 12 weeks to prepare heart sections. The hiPSC-CM grafts were trimmed and collected from the tissue sections via LCM. Gene expression in the hiPSC-CM grafts was evaluated by RNA sequencing (Figure 2A). Human-derived mRNAs were distinguished from mouse-derived mRNAs and only the former was analyzed. Gene expression differed between the early (2 and 4 weeks after transplantation) and late (12 weeks after transplantation) groups (Figure 2B). Moreover, PC2 in the principal component analysis separated the samples into the two mentioned groups (Figure 2C). Gene ontology analysis showed that the expression of genes related to blood vessel and cardiovascular system development was upregulated in the late group compared with that in the early group (Figure 2D). Conversely, the expression of genes related to metabolic processes and cell cycle was downregulated in the late group compared with that in the early group (Figure 2E). Next, we generated a heatmap of the expression of characteristic genes (Figure 2F). Genes that were highly expressed in mature CMs, such as *MYH7*, *TNNI3*, and *MYL2*, were upregulated over time after transplantation. Conversely, the expression of genes that were highly expressed in immature CMs, such as *MYH6*, *TNNI1*, and *MYL7*, was downregulated. The gene expression of some ion channels, such as *KCNJ2*, which is involved in the formation of the resting membrane potential, was upregulated in the late group. Furthermore, the expression of genes related to transverse tubules, such as *BIN1*, *CAV1*, *CAV2*, and *CAV3*, was also upregulated. Next, we focused on metabolism, and the expression of genes related to glycolysis, such as *SLC2A1*, *HK2*, and *LDHA*, was downregulated in the late group compared with that in the early group. Conversely, the expression of genes related to fatty acid transport, such as *CD36*, and FAO, such as *PPARA* and *PPARD* (the genes of peroxisome proliferator-activated receptor [PPAR]), was upregulated. Since CM metabolism switches from glycolysis to FAO during fetal and neonatal stages,^32^^,^^33^ the present results indicated that hiPSC-CMs underwent metabolic maturation *in vivo*.

Further, we performed IMS on the heart sections containing the hiPSC-CM grafts and measured the levels of various metabolites in the grafts and host myocardium (Figure 2A). The ratio of the average signal intensity between the transplanted hiPSC-CM grafts and the host myocardium was calculated (Table S1). Representative data from each time period showing hexose diphosphate accumulation is presented in Figure 2G. Hexose diphosphate accumulated 4.00 times more in the hiPSC-CM graft than in the host myocardium 2 weeks after transplantation. The ratios were 2.11 times at 4 weeks and 1.14 times at 12 weeks. We measured the ratio of the signal intensity of some glycolytic metabolites between the hiPSC-CM grafts and the host myocardium over time (Figure 2H). Phosphoenolpyruvic acid (PEP) and 2-phosphoglycerate (2PG)/3-phosphoglycerate (3PG) accumulation in the hiPSC-CM grafts 2 weeks after transplantation was significantly higher than that in the host myocardium. Their accumulation in the hiPSC-CM grafts decreased over time, particularly, the accumulation of 2PG/3PG significantly decreased from 2 weeks to 12 weeks. A similar tendency was observed for hexose diphosphate; the accumulation in the hiPSC-CM grafts was higher than that in the host myocardium at 2 weeks while not significantly, but significantly decreased from 2 weeks to 12 weeks. These results suggested that hiPSC-CMs in the grafts actively underwent glycolysis for energy production immediately after transplantation; however, their dependence on glycolysis gradually decreased. The accumulation of the metabolites of the tricarboxylic acid (TCA) cycle and amino acids in hiPSC-CM grafts did not change over time (Figure S2A, S2D, and S2E). Generally, the acylcarnitine amounts reflect the FAO activity.^49^ Although RNA sequencing of the hiPSC-CM grafts showed that the expression of genes related to fatty acid transport and FAO was upregulated over time after transplantation, there was no significant difference in the accumulation of acylcarnitines, such as tetradecanoylcarnitine, palmitoylcarnitine, and stearoylcarnitine, between the hiPSC-CM grafts and host myocardium and between the hiPSC-CM grafts at each time period (Figure S2B and S2C).

Based on the results of RNA sequencing and IMS, the energy production pathway of the transplanted hiPSC-CM grafts switched from glycolysis to FAO. Hence, the hiPSC-CM grafts undergo the process of metabolic maturation after transplantation.

### hiPSC-Ts showed high gene expression of amino acid transporters and high levels of accumulated amino acids

**Figure 3:**
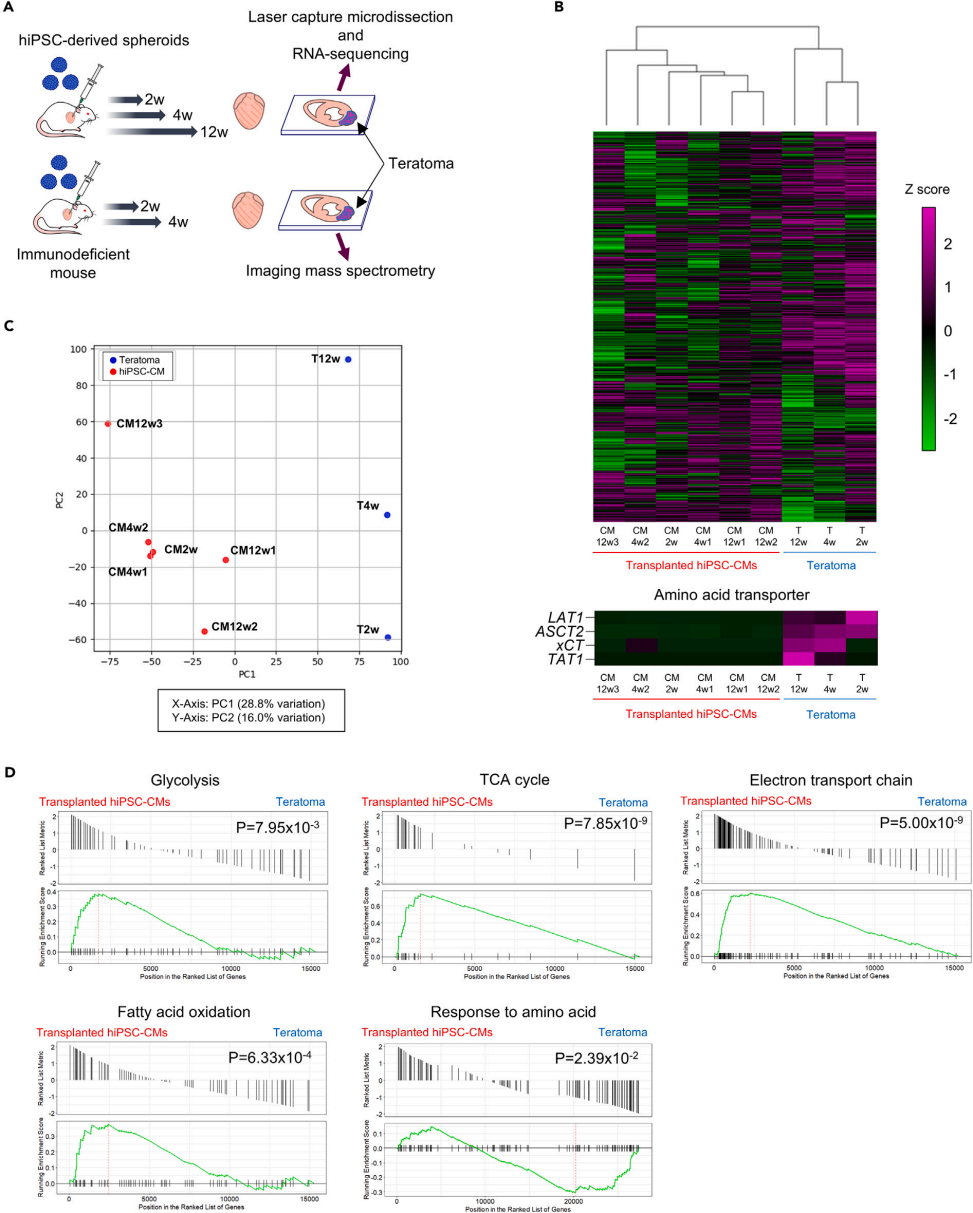
Metabolic differences between hiPSC-CM and -T grafts (A) Scheme of the experiment. (B) Upper: heatmap showing the results of RNA sequencing on hiPSC-CM and hiPSC-T grafts. Lower: heatmap showing the gene expression of amino acid transporters. (C) Principal-component analysis of hiPSC-CM (red) and -T (blue) grafts at 2, 4, and 12 weeks. (D) Gene set enrichment analysis demonstrating differences in gene expression between hiPSC-CM and -T grafts.

Undifferentiated hiPSCs were cultured in microwells for 1 day to obtain hiPSC spheroids, which were then transplanted into the hearts of NOG mice to obtain hiPSC-Ts (Figure 3A). Immunohistochemistry showed that hiPSC-Ts contained many vimentin-positive cells, suggesting that hiPSC-Ts were mainly mesenchymal tissue (Figure S3A). hiPSC-Ts were trimmed and collected from the tissue sections via LCM. The gene expression of hiPSC-Ts at 2, 4, and 12 weeks after transplantation was evaluated by RNA sequencing and compared with that of the hiPSC-CM grafts evaluated in the same manner (Figures 3B and 3C). To evaluate differences in gene expression between hiPSC-CM and -T grafts, we performed gene set enrichment analysis (GSEA) (Figure 3D). The expression of genes related to glycolysis, the TCA cycle, the electron transport chain (ETC), and FAO was lower in the hiPSC-T grafts than that in the hiPSC-CM grafts. Conversely, the expression of genes related to the response to amino acids was higher in the hiPSC-T grafts than that in the hiPSC-CM grafts. Furthermore, we generated heatmaps of the expression of characteristic genes related to glycolysis, the TCA cycle, the ETC, and FAO (Figure S3A). The gene expression of amino acid transporters was higher in the hiPSC-T grafts than that in the hiPSC-CM grafts (Figure 3B). Specifically, *LAT1* and *ASCT2* expression was 20.10 times and 33.42 times higher in the hiPSC-T grafts than in the hiPSC-CM grafts.

**Figure 4:**
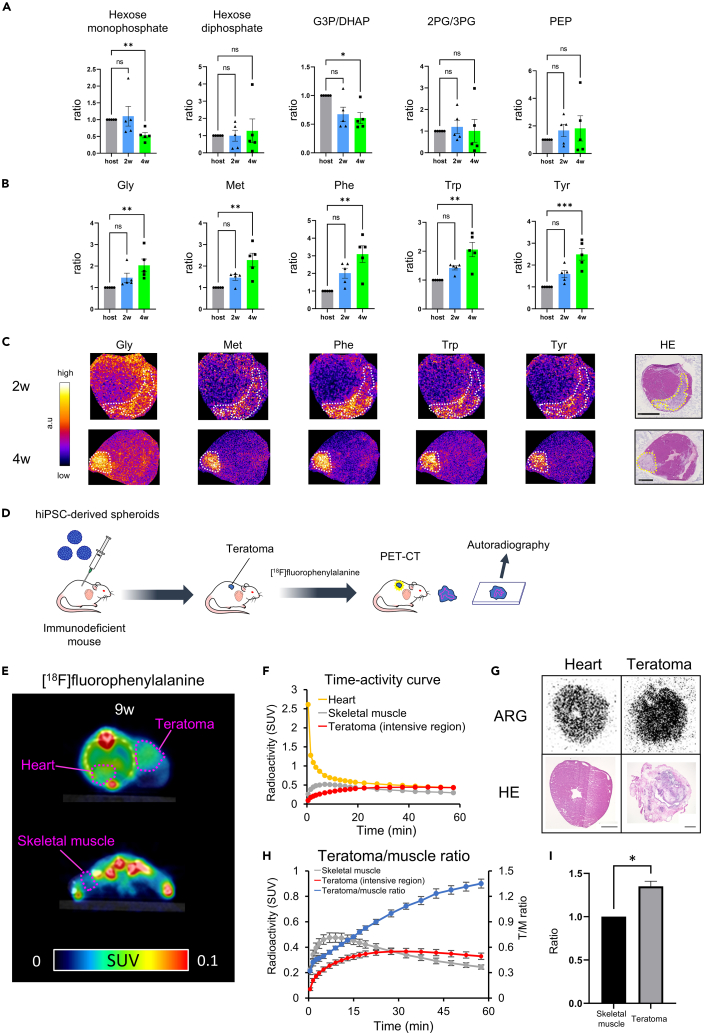
Metabolism of hiPSC-Ts evaluated via IMS and PET imaging (A) The ratio of accumulation of glycolytic metabolites between hiPSC-Ts and the host myocardium measured by IMS (*n* = 5, respectively. Dunn’s multiple comparisons test. ∗*p* < 0.05. Error bars represent SEM). G3P/DHAP: glyceraldehyde-3-phosphate/dihydroxyacetone phosphate. 2PG/3PG, 2-phosphoglycerate/3-phosphoglycerate; PEP, phosphoenolpyruvic acid. (B) The ratio of accumulation of amino acids between hiPSC-Ts and the host myocardium measured by IMS (*n* = 5, respectively. Dunn’s multiple comparisons test. ∗*p* < 0.05. Error bars represent SEM). (C) Representative data showing the accumulation of amino acids in hiPSC-Ts and the host myocardium measured by IMS. (D) Scheme of the PET imaging experiment using [^18^F]fPhe. (E) Representative PET image of the subcutaneous hiPSC-T by [^18^F]fPhe 9 weeks after transplantation (40–60 min, integrated images). (F) Time-activity curve expressing the radioactivity of [^18^F]fPhe in each organ. (G) Autoradiography of the heart and subcutaneous hiPSC-T graft. (H) Time-activity curve expressing the mean values of the radioactivity of [^18^F]fPhe in hiPSC-Ts and in skeletal muscles and the tumor/muscle ratio (*n* = 6. Error bars represent SEM). (I) The mean values of the radioactivity of [^18^F]fPhe in hiPSC-Ts and skeletal muscles around 60 min (*n* = 6. One-sample Wilcoxon signed rank test. ∗*p* < 0.05. Error bars represent SEM).

We analyzed metabolites in hiPSC-Ts by performing IMS 2 and 4 weeks after transplantation (Table S2). Among glycolytic metabolites, hexose monophosphate and glyceraldehyde-3-phosphate/dihydroxyacetone phosphate accumulation in the hiPSC-Ts was significantly lower than that in the host myocardium 4 weeks after transplantation, and their ratio was about half (Figure 4A). No significant differences were observed for the levels of hexose diphosphate, 2PG/3PG, and PEP between hiPSC-Ts and the host myocardium. Additionally, the accumulation of TCA cycle metabolites and acylcarnitines did not differ between hiPSC-Ts and the host myocardium (data not shown). Further, the signal intensities of amino acids, such as glycine (Gly), Met, tryptophan (Trp), phenylalanine, (Phe) and tyrosine (Tyr), in hiPSC-Ts were significantly higher than those in the host myocardium at 4 weeks, and their ratios were greater than 2.00 (Figures 4B and 4C). Considering the accumulation of these amino acids and the higher gene expressions of amino acid transporters in hiPSC-Ts, it is possible that these amino acids are taken up more in hiPSC-Ts.

### [^18^F]fluorophenylalanine detected subcutaneous hiPSC-Ts

Based on the results of RNA sequencing and IMS, we investigated a method for detecting hiPSC-Ts using PET imaging. First, hiPSC spheroids were transplanted into the hearts of NOG mice and subjected to [^18^F]FDG-PET 4 and 12 weeks after transplantation (Figure S4A). However, [^18^F]FDG-PET could not visualize hiPSC-Ts in the heart, which is consistent with the previous study^44^ (Figure S4B). After PET imaging, the blood was washed out by perfusion with saline. Then, the heart sections were prepared and subjected to autoradiography. The results showed that [^18^F]FDG uptake by hiPSC-Ts was lower than that by the host myocardium (Figure S4C). Secondly, we focused on PET imaging using amino acids. [^11^C]Met-PET was performed because Met was one of the amino acids that accumulated in hiPSC-Ts. We transplanted hiPSC spheroids into the heart and subcutaneous tissue of NOG mice and performed [^11^C]Met-PET 12 weeks after transplantation (Figure S4A). One mouse had a hiPSC-T in the heart and the other had hiPSC-Ts in the heart and subcutaneous tissues. As a result, the radioactivity of the hiPSC-Ts was extremely low, and [^11^C]Met-PET could not visualize hiPSC-Ts (Figure S4D). Autoradiography showed that [^11^C]Met uptake in the intracardiac and subcutaneous hiPSC-Ts was lower than that in the host myocardium (Figure S4E).

Next, we focused on Phe, which had the highest accumulation in hiPSC-Ts among amino acids. We transplanted hiPSC spheroids into the subcutaneous tissues of NOG mice and administered [^18^F]fluorophenylalanine ([^18^F]fPhe) 9 weeks after transplantation (Figure 4D). Representative data from [^18^F]fPhe-administered mice are shown in Figures 4E–4G. Radioactivity was measured over time until 60 min after the administration. We were able to visualize the subcutaneous hiPSC-T by performing [^18^F]fPhe-PET (integrated images were obtained 40–60 min after administration) (Figure 4E). The radioactivity of the subcutaneous hiPSC-T increased gradually. The ratio of radioactivity between the subcutaneous hiPSC-T and the skeletal muscle (teratoma/muscle ratio) increased by 1.57-folds around 60 min (Figure 4F). After PET imaging, autoradiography was performed. Autoradiography is not affected by the PET tracer in the blood and can evaluate the radioactivity of the tracer taken into the tissues. As a result, [^18^F]fPhe uptake in the hiPSC-T was higher than that in the host myocardium (Figure 4G). Further, we analyzed the radioactivity of hiPSC-Ts and skeletal muscles in six mice that were administered with [^18^F]fPhe including the mouse presented previously (Figures 4E and S4F). The teratoma/muscle ratio increased over time (Figure 4H). The radioactivity of the hiPSC-Ts was significantly higher than that of the skeletal muscles around 60 min, and the ratio reached 1.35-folds (Figure 4I). hiPSC-Ts that formed ectopically, such as in subcutaneous tissues or skeletal muscles, could be visualized by [^18^F]fPhe.

## Discussion

Herein, we present the metabolic profiles of hiPSC-CM and -T grafts after the transplantation of hiPSC-CSs and undifferentiated hiPSC spheroids. To the best of our knowledge, this is the first study in which IMS has been performed on sections of hiPSC-CM and -T grafts.

We evaluated the morphological maturation of the present hiPSC-CM grafts by performing immunohistochemical experiments (Figure 1). The sarcomere length of hiPSC-CMs increased over time *in vivo* and reached around 1.8 μm 6 months after transplantation. Generally, the sarcomere length of adult CMs was approximately 1.7–2.2 μm.^50^ Thus, we could not conclude whether the transplanted hiPSC-CMs were mature enough at 6 months of transplantation. Further maturation may occur if they are observed for a longer time or if they are transplanted into human hearts as allotransplants. At 6 months, all transplanted hiPSC-CMs were MLC2v-positive/MLC2a-negative and Ki67-negative, indicating that they were mature enough to be referred to as ventricular CMs. Several *in vitro* studies have been performed to elucidate the mechanism underlying hiPSC-CM maturation.^16^^,^^17^^,^^18^^,^^19^^,^^20^^,^^21^^,^^22^^,^^27^^,^^28^^,^^29^^,^^51^, ^52^, ^53^, ^54^ Herein, we speculated that hiPSC-CM maturation *in vivo* was driven by mechanical stretch, electrical stimulation, extracellular matrix, paracrine effects from the surrounding CMs and non-CMs, such as endothelial and epicardial cells, and endocrine effects of hormones.

Next, we evaluated metabolic changes in these hiPSC-CMs after transplantation by performing RNA sequencing and IMS (Figure 2). The expression of genes related to glycolysis was downregulated, whereas the expression of genes related to fatty acid transport, such as *CD36*, and FAO, such as *PPARA* and *PPARD*, was upregulated. Specifically, PPARdelta signaling was related to the functional and metabolic maturation of hPSC-CMs.^25^ The accumulation of some glycolytic metabolites in the hiPSC-CM grafts was higher than that in the host myocardium 2 weeks after transplantation; however, this accumulation decreased over time to the same level as that in the host myocardium. The metabolism of fetal CMs depends on glycolysis, whereas that of CMs in the late neonatal and subsequent phases mainly depends on FAO.^32^^,^^33^ Therefore, these results suggested that the transplanted hiPSC-CMs switched from glycolysis to FAO and underwent metabolic maturation. Nevertheless, the accumulation of acylcarnitines, which reflects the activity of fatty acid metabolism,^49^ was generally lower in the hiPSC-CM grafts than that in the host myocardium. Hence, the metabolic maturation of the transplanted hiPSC-CMs at 12 weeks may not have reached the same level as that in the host myocardium.

Next, we evaluated hiPSC-T metabolism (Figures 3 and 4). We performed RNA sequencing of hiPSC-Ts and found that the expression of genes related to glycolysis, TCA cycle, ETC, and FAO was downregulated, whereas the gene expression of amino acid transporters was upregulated. Moreover, the IMS results showed that many amino acids accumulated in these hiPSC-Ts.

Based on these findings, we examined whether hiPSC-Ts could be detected by using [^18^F]FDG and PET tracers derived from amino acids (Figure 4). [^18^F]FDG uptake by hiPSC-Ts was low and could not be detected. This result was in line with the low expression of genes related to glycolysis in hiPSC-Ts. Clinically, an ovarian teratoma can be detected by [^18^F]FDG if malignant, but cannot if benign.^22^ hiPSC-Ts are also considered to be undetectable by [^18^F]FDG unless malignant transformation occurs.

Although amino acid metabolism in malignant tumors has been well-studied, information on amino acid uptake and utilization by hiPSC-Ts is scarce. Amino acids are important for the survival and growth of malignant tumors and are used as energy sources and building blocks for proteins.^55^ Amino acid transporters, such as LAT1 and ASCT2, are highly expressed in the cancer cell membrane.^56^ LAT1 transports Met, Phe, Trp, Tyr, leucine, isoleucine, valine, and histidine from outside the cell to the cytoplasm, whereas it transports Gln from the cytoplasm to outside the cell as an exchange substrate.^57^ ASCT2 exhibits the Na^+^-dependent uptake of Gln, Cys, alanine, serine, and threonine into the cytoplasm.^58^ These amino acid transporters were highly expressed in hiPSC-Ts, and Gly, Met, Phe, Tyr, and Trp accumulated in the hiPSC-Ts more than twice as much as in the host myocardium. [^11^C]Met is the most common tracer of amino acid tracers.^59^ In this study, intracardiac and subcutaneous hiPSC-Ts had little uptake of [^11^C]Met. We then performed [^18^F]fPhe-PET because Phe was the most accumulated amino acid in the hiPSC-T grafts. [^18^F]fPhe uptake in hiPSC-Ts was significantly higher than that in skeletal muscles around 60 min after administration. Therefore, it was suggested that hiPSC-Ts formed ectopically, such as in skeletal muscles and subcutaneous tissues, are detected using [^18^F]fPhe. Originally, Hoyte et al. synthesized [^18^F]fPhe and evaluated its biodistribution.^60^ Subsequently, it has been used to study cerebral protein synthesis^61^ and detect brain tumors.^62^^,^^63^^,^^64^ [^18^F]fPhe is incorporated into cells via L-type amino acid transporters. LAT1 is a type of L-type amino acid transporter that is specifically expressed in cancer cells.^57^ It is possible that hiPSC-Ts could be detected more sensitively using amino acid-derived tracers with higher LAT1 specificity.^65^^,^^66^^,^^67^^,^^68^

In this study, we revealed that hiPSC-CM grafts undergo the process of metabolic maturation after transplantation. Indeed, glycolysis was active in the hiPSC-CM grafts immediately after transplantation, but gradually shifted to FAO. We also examined the metabolic profiles of hiPSC-Ts. Upregulated gene expression of amino acid transporters and high accumulation of amino acids, such as Met and aromatic amino acids, were observed in hiPSC-Ts. Then we demonstrated that subcutaneous hiPSC-Ts could be detected *in vivo* using [^18^F]fPhe. These results provided insights for the clinical application of cardiac regenerative therapy.

### Limitations of the study

The limitation of this study is that we did not perform experiments using a myocardial infarction model. Further studies are expected to reveal how hiPSC-CMs mature metabolically in an ischemic environment because cell transplantation therapy using hiPSC-CMs is intended to be clinically used for patients with myocardial infarction. Moreover, it would be desirable to perform experiments on hiPSC-Ts using a myocardial infarction model as well. However, as the detailed metabolic profiles of transplanted hiPSC-CMs and -Ts have not been evaluated *in vivo* even in a healthy model in previous studies, we decided to use a healthy model as the first step.

Our group purifies hiPSC-CMs using glucose- and glutamine-free medium supplemented with lactate.^5^^,^^6^ This selection method switches the metabolism of hiPSC-CMs from glycolysis to oxidative phosphorylation, inducing their structural and metabolic maturation.^69^ Therefore, it can be inferred that hiPSC-CMs selected by this method tend to engraft and mature in ischemic regions, where glucose is depleted and lactate accumulates.^70^^,^^71^ Indeed, our group has already transplanted hiPSC-CSs to cynomolgus monkeys with myocardial infarction and showed large engraftment and morphological maturation of hiPSC-CMs.^12^ The results of metabolic maturation obtained in the healthy model in this study will be useful in considering the metabolism of hiPSC-CMs in an ischemic environment.

## Resource availability

### Lead contact

Further information regarding the resources in this study should be directed to and will be provided by the lead contact, Shugo Tohyama (shugo.tohyama@fujita-hu.ac.jp).

### Material availability

This study did not generate new unique material and reagents.

### Data and code availability

• The data of RNA sequencing generated during this study have been deposited in the Gene Expression Omnibus (GEO). The accession number is GSE: GSE279695.
• This paper does not report original code.
• Any additional information required to reanalyze the data reported in this paper is available from the lead contact upon request.

## Acknowledgments

The authors thank Sayaka Kanaami, Tomoko Haruna, Rei Ohno, Kuniko Momoi, Miho Yamaguchi, Yuki Yamamoto, Yui Narita, Naoko Matsumoto, and Yoshiko Miyake for technical assistance with cell preparation, cell culture, and tissue preparation (Department of Cardiology, Keio University) and Katsushi Kumata, Nobuki Nengaki, Masanao Ogawa, Hidekatsu Wakizaka, and Yiding Zhang for radiosynthesis and PET operations (National Institutes for Quantum Science and Technology). The authors also thank Dr. Masahiro Jinzaki, Dr. Yasuhisa Fujibayashi, and Dr. Takehiro Nakahara for their advice and support throughout the PET studies, and CiRA for providing the hiPSC lines (253G4 and Ff-I14). The authors thank DNA Chip Research Inc. for the RNA sequencing. This work was supported by the 10.13039/501100001691Japan Society for the Promotion of Science KAKENHI (grants 23K19571, 24K11198 to Y.S., 23H02992 to S.Tohyama, and 19H03660 to J.F.), an 10.13039/100009619AMED grant (24bm1123010 to S. Tohyama), a Grant-in-Aid for 10.13039/501100001691JSPS Fellows (21J10680 to Y.S.), the 10.13039/501100005072Japanese Circulation Society (to S. Tohyama), 10.13039/100018456KISTEC (to S. Tohyama), and Heartseed, Inc.

## Author contributions

S. Tohyama conceptualized and designed the study; Y.S. performed and analyzed most experiments; N.K. performed cell preparation, animal breeding, and assistance with cell transplantation; A.K. performed IMS; T.Y. performed PET imaging; the other co-authors contributed to specific experiments; Y.S. and S. Tohyama wrote the original draft; S. Tohyama, H.T., T.C.U., S. Tanosaki, and S.S. reviewed and edited the manuscript; Y.S., S. Tohyama, J.F., and K.F. acquired funding; and S. Tohyama, J.F., M.-R.Z., M.S., K.F., and M.I. supervised the study.

## Declaration of interests

K.F. is the co-founder and CEO of Heartseed Inc. S.Tohyama is an advisor from Heartseed Inc. S. Tohyama, H.K., J.F., and K.F. own equity in Heartseed Inc.

## STAR★Methods

### Key resources table

**Table:** 

REAGENT or RESOURCE	SOURCE	IDENTIFIER
**Antibodies**		

Anti-cardiac troponin I	Abcam	Cat#ab52862; RRID:AB_869983
Anti-troponin T, cardiac isoform Ab-1 (Clone 13-11)	Thermo Fisher Scientific	Cat#MS-295-P; RRID:AB_61806
Anti-cardiac troponin T (clone: REA 400)	Miltenyi Biotec	Cat#130-129-225; RRID:AB_2922012
Anti-α actinin	Abcam	Cat#ab137346; RRID:AB_2909405
Anti-myosin light chain 2a (MLC2a)	Synaptic Systems	Cat#SYSY 311-011; RRID:AB_2266770
Anti-myosin light chain 2v (MLC2v)	Abcam	Cat#ab79935; RRID:AB_1952220
Anti-human nuclei	Merck Millipore	Cat#MAB1281; RRID:AB_94090
Anti-CD31	Abcam	Cat#ab28364; RRID:AB_726362
anti-proliferating cell protein Ki-67	Sigma-Aldrich	Cat#P6834; RRID:AB_261141
Alexa Fluor® 594 donkey anti-rabbit IgG	Invitrogen	Cat#A21207; RRID:AB_141637
Alexa Fluor® 488 donkey anti-mouse IgG	Invitrogen	Cat#A21202; RRID:AB_141607
Alexa Fluor® 488 donkey anti-goat IgM	Invitrogen	Cat#A21042; RRID:AB_141357

**Chemicals, peptides, and recombinant proteins**		

Hoechst® 33342	Thermo Fisher Scientific	Cat#H3570
CHIR99021	FUJIFILM Wako Pure Chemical	Cat#034-23103
BMP4	R&D Systems	Cat#314-BP
IWR-1	Sigma-Aldrich	Cat#I0161
Y-27632	FUJIFILM Wako Pure Chemical	Cat#034-24024
Orlistat	Sigma-Aldrich	Cat#O4139
Matrigel	CORNING	Cat#354230
Vitronectin	Thermo Fisher Scientific	Cat#A14700
Dulbecco’s phosphate-buffered saline (D-PBS)	FUJIFILM Wako Pure Chemical	Cat#045-29795
AS103C	Ajinomoto	N/A
RPMI-1640	FUJIFILM Wako Pure Chemical	Cat#189-02025
Minimum Essential Medium Eagle (MEM)-α	Thermo Fisher Scientific	Cat#12571-048
AS501	Ajinomoto	N/A
B27 supplement without insulin	Thermo Fisher Scientific	Cat#A1895601
Fetal bovine serum (FBS)	Biowest	Cat#S1560-500
Sodium pyruvate	Sigma-Aldrich	Cat#S8636
TrypLE Select	Thermo Fisher Scientific	Cat#12563-011
2.5g/L-Trypsin/1mmol/L-Ethylenediaminetetraacetic acid (EDTA)	NACALAI TESQUE	Cat#35554-64
CryoStor CS10	STEMCELL Technologies	Cat#ST-07931
TRIzol Reagent	Life Technologies	Cat#15596026
9-aminoacridine	Merck Millipore	Cat#818362
α-cyano-4-hydroxycinnamic acid	Sigma-Aldrich	Cat#476870

**Critical commercial assays**		

miRNeasy Micro Kit	QIAGEN	Cat#217084
SMART-Seq Stranded Kit	Clontech	Cat#634442

**Experimental models: Cell lines**		

Ff-I14 hiPSCs	Provided by CiRA at Kyoto University	N/A
253G4 hiPSCs	Provided by CiRA at Kyoto University	N/A

**Experimental models: Organisms/strains**		

NOD/Shi-scid IL-2RγKO (NOG) mouse	*In Vivo* Science Inc.	N/A

**Software and algorithms**		

ImageJ	Rasband et al.^72^	https://imagej.net/ij/index.html
GraphPad Prism 9	Graphpad	Cat#GPPEAC
STAR 2.7.5c	Dobin et al.^73^	https://github.com/alexdobin/STAR
StrandNGS 3.4	Agilent Technologies	N/A
Python3 [version 3.8]	Von Rossum et al.^74^	https://www.python.org/
R [version 4.2.2]	R Core Team^75^	https://www.R-project.org/
pandas [version 1.2.4]	McKinney^76^	https://pypi.org/project/pandas/1.2.4/
matplotlib [version 3.2.1]	Hunter^77^	https://matplotlib.org/
gplots [version 3.1.3]	Warnes et al.^78^	https://github.com/talgalili/gplots
numpy [version 1.18.5]	Harris et al.^79^	https://numpy.org/ja/
sklearn [version 0.23.1]	Pedregosa et al.^80^	https://scikit-learn.org/stable/
org.Hs.e.g.,.db [version 3.16.0]	Carlson^81^	https://bioconductor.org/packages/release/data/annotation/html/org.Hs.eg.db.html
DOSE [version 3.24.2]	Yu et al.^82^	https://yulab-smu.top/biomedical-knowledge-mining-book/
ClusterProfiler [version 4.2.2]	Wu et al.^83^	https://yulab-smu.top/contribution-knowledge-mining/
IMAGEREVEAL™ MS software	Shimadzu Corp	N/A
Nanozoomer-XR	Hamamatsu Photonics	N/A
PMOD software	PMOD Technologies	N/A

**Other**		

15 cm dish coated with collagen type I	AGC TECHNO GLASS	Cat#4030-010
Elplasia multiwell plate	CORNING	Cat#4440

### Experimental model and study participant details

HiPSC lines, 253G4 and Ff-I14, were provided by the Center for iPS Cell Research and Application, Kyoto University. The cells were cultured in a humidified 5% CO_2_ incubator at 37°C and routinely tested for mycoplasma contamination. HiPSCs were maintained in the modified StemFit medium AS103C (Ajinomoto, Tokyo, Japan).^7^^,^^10^^,^^11^ The cells were passaged every 4–7 days. Upon passaging, they were washed with Dulbecco’s phosphate-buffered saline (D-PBS) (FUJIFILM Wako Pure Chemical, Osaka, Japan, 045–29795) and incubated for 3 min with TrypLE Select (Thermo Fisher Scientific, Waltham, MA, USA, 12563-011) at 37°C. The cells were collected in AS103C supplemented with 10 μM Y-27632 (FUJIFILM Wako Pure Chemical, Osaka, Japan, 034–24024) and pelleted for 3 min at 300 rcf. Then, the cells were resuspended and counted using Vi-CELL (Beckman Coulter, Brea, CA, USA). Next, the cells were plated in dishes coated with Matrigel (CORNING, Corning, NY, USA, 354230).

Adult male NOG mice (*In Vivo* Science Inc., Tokyo, Japan) were purchased. The experimental protocol was approved by the Sub-Committee on Animal Care and the Institutional Review Board of Keio University and the National Institutes for Quantum and Radiological Science and Technology according to the Fundamental Guidelines for Proper Conduct of Animal Experiments and Related Activities in Academic Research Institutions (Ministry of Education, Culture, Sports, Science, and Technology). All the animals received humane care in accordance with the Guide for the Care and Use of Laboratory Animals.

### Method details

#### Differentiation of human induced pluripotent stem cells into CMs

On day 0, hiPSCs were rinsed with D-PBS and incubated with Roswell Park Memorial Institute (RPMI)-1640 (FUJIFILM Wako Pure Chemical, Osaka, Japan, 189–02025) supplemented with 2% B27 supplement without insulin (Thermo Fisher Scientific, Waltham, MA, USA, A1895601), 6 μM CHIR99021 (FUJIFILM Wako Pure Chemical, Osaka, Japan, 034–23103), and 1 ng/mL bone morphogenic protein 4 (R&D Systems, Minneapolis, MN, USA, 314-BP) for 1 day. On day 1, the cells were rinsed with D-PBS and incubated with RPMI-1640 supplemented with B27 supplement without insulin. On day 3, the cells were rinsed with D-PBS and incubated with RPMI-1640 supplemented with B27 supplement without insulin and 5 μM IWR-1 (Sigma-Aldrich, St. Louis, MO, USA, I0161). On day 6, the cells were rinsed with D-PBS and incubated with RPMI-1640 supplemented with B27 supplement without insulin. On day 7, the cells were incubated in Minimum Essential Medium Eagle (MEM)-α (Thermo Fisher Scientific, Waltham, MA, USA, 12571-048) supplemented with 5% fetal bovine serum (FBS) (Biowest, Nuaillé, France, S1560-500) and 2 mM sodium pyruvate (Sigma-Aldrich, St. Louis, MO, USA, S8636). On day 10, the cells were rinsed with D-PBS and incubated for 5 min with 0.25% trypsin/ethylenediaminetetraacetic acid (EDTA) (NACALAI TESQUE, Kyoto, Japan, 35554-64). Then, the cells were collected in MEM-α supplemented with 5% FBS and 2 mM sodium pyruvate and pelleted for 3 min at 300 rcf. Next, the cells were resuspended in MEM-α supplemented with 5% FBS, 2 mM sodium pyruvate, and 6 μM Orlistat (Sigma-Aldrich, St. Louis, MO, USA, O4139) and counted using Vi-CELL.^8^^,^^9^ The cells were plated in dishes coated with collagen type I (AGC TECHNO GLASS, Shizuoka, Japan, 4030-010). On day 13, the culture medium was replaced with glucose- and glutamine-free medium supplemented with 4 mM lactate, the StemFit medium AS501 (Ajinomoto, Tokyo, Japan).^5^^,^^6^ On days 14–16, the medium was replaced daily with the AS501 medium. On day 17, the cells were incubated for 3 min with D-PBS and then incubated for 5 min with the 0.25% trypsin/EDTA solution. The cells were collected in MEM-α supplemented with 5% FBS and 2 mM sodium pyruvate and pelleted for 3 min at 300 rcf. The cells were dispensed into microtubes for cryopreservation using CryoStor CS10 (STEMCELL Technologies, Vancouver, Canada, ST-07931).

#### Production of hiPSC-derived cardiac spheroids and human induced pluripotent stem cell spheroids

Differentiation efficiency and purity were evaluated via the flow cytometric analysis of cardiac troponin T levels using a troponin T antibody (clone: REA400; Miltenyi Biotec, Auburn, CA, USA, 130-129-225). HiPSC-CSs were composed of pure hiPSC-CMs in Elplasia multiwell plates (CORNING, Corning, NY, USA, No 4440 or Kuraray, Tokyo, Japan, 400 560 CL6) for a week as previously reported.^35^ One cardiac spheroid contained ∼1,000 CMs, and its size was ∼200 μm. Similarly, hiPSC spheroids were incubated with hiPSCs in the microwell plates for 1 day.

Ff-I14 hiPSC-CSs were used for experiments in which maturation of hiPSC-CM grafts after transplantation was assessed using immunohistochemistry. G4 hiPSC-CSs and G4 hiPSC-spheroids were used for experiments in which metabolic profiles of hiPSC-CM and -T grafts were evaluated using RNA sequencing and IMS. Finally, G4 hiPSC-spheroids were used for experiments of PET imaging.

#### Transplantation of hiPSC-derived cardiac spheroids and human induced pluripotent stem cell spheroids into the myocardium and subcutaneous tissue of NOG mice

For transplantation of hiPSC-CSs or hiPSC spheroids into the heart of the NOG mice, the mice were anesthetized with a low dose of isoflurane. The anesthetized mice were ventilated using a rodent ventilator (Shinano, Tokyo, Japan, SN-480-7). A left thoracotomy was performed in the fourth intercostal space. HiPSC-CSs or hiPSC spheroids (1 × 10^6^–3 × 10^6^ cells) were transplanted into the myocardium with 60 μL PBS by using 27G syringes (Terumo, Tokyo, Japan, SS-10M2713). Then, intercostals and skin were sutured by nylon sutures (Natsume, Tokyo, Japan, AR15-40N3).

For transplantation of hiPSC spheroids into the subcutaneous tissues, the mice were anesthetized with a low dose of isoflurane. HiPSC spheroids (3 × 10^7^–4 × 10^7^ cells) were transplanted into the subcutaneous tissue of the back with 300 μL of 1:1 mixture of PBS and Matrigel, or PBS and vitronectin (Thermo Fisher Scientific, Waltham, MA, USA, A14700) by using 27G syringes.

#### Immunohistochemistry

Frozen sections were fixed with 4% paraformaldehyde, and formalin-fixed paraffin-embedded sections were prepared. The sections were stained with the following antibodies: Primary antibodies used for hiPSC-CMs and hiPSC-Ts were cardiac-TnI (Abcam, Cambridge, MA, USA, ab52862), troponin T, cardiac isoform Ab-1(Clone 13-11) (Thermo Fisher Scientific, Waltham, MA, USA, MS-295-P), α-actinin antibody (Abcam, Cambridge, MA, USA, ab137346), myosin light chain 2a (MLC2a) (Synaptic Systems, Göttingen, Germany, SYSY 311-011), myosin light chain 2v (MLC2v) (Abcam, Cambridge, USA, ab79935), anti-human nuclei (Merck Millipore, Burlington, MA, USA, MAB1281), anti-CD31 (Abcam, Cambridge, USA, ab28364), and monoclonal anti-proliferating cell protein Ki-67 (Sigma-Aldrich, St. Louis, MO, USA, P6834). Secondary antibodies were Alexa Fluor 594 donkey anti-rabbit IgG (Invitrogen, Waltham, MA, USA, A21207), Alexa Fluor 488 donkey anti-mouse IgG (Invitrogen, Waltham, MA, USA, A21202), and Alexa Fluor 488 donkey anti-goat IgM (Invitrogen, Waltham, MA, USA, A21042). The nuclei were stained with Hoechst 33342 (Thermo Fisher Scientific, Waltham, MA, USA, H3570). All images were acquired and analyzed using a microscope (BIOREVO, BZ-X710, Keyence, Osaka, Japan) and ImageJ.^72^

#### Laser capture microdissection

The hearts were embedded in an optimal cutting temperature (OCT) compound (Tissue-Tek, SAKURA, Tokyo, Japan, 4583) or Super Cryoembedding Medium (SCEM, Section-Lab Co. Ltd., Japan). The samples were then placed on dry ice. The frozen heart tissues were sectioned at a thickness of 7 μm using Cryostat (Leica, Wetzlar, Germany) and placed on membrane-coated slides. These sections were fixed with 95% ethanol for 2 min. Then, they were stained with 0.1% toluidine blue to detect hiPSC-CM or -T grafts. The hiPSC-CM or -T grafts were captured and placed in tubes using an LCM system (PALM MB-IV, Zeiss, Oberkochen, Germany). Then, 350 μL of TRIzol Reagent (Life Technologies, Carlsbad, CA, USA, 15596026) was added to each tube. The samples were then vortexed for 30 min.

#### RNA sequencing

Total RNA was extracted using miRNeasy Micro Kit (QIAGEN, Hilden, Germany). Complementary DNA was synthesized using SMART-Seq Stranded Kit (Clontech, Mountain View, CA, USA). Library preparations were sequenced using NextSeq500 (Illumina, San Diego, CA, USA). Combined data of human (hg38) and mouse (mm10) genome were organized in silico. The reads were mapped using STAR 2.7.5c (GitHub, San Francisco, CA, USA).^73^ The proportion of the reads assigned to the hg38 or mm10 reference was counted. The reads assigned to hg38 were classified as human reads, and the alignment files (BAM) were created.^84^ The aligned reads were subjected to downstream analyses using the StrandNGS 3.4 software (Agilent Technologies, Inc., CA, USA). The read counts allocated for each gene and transcript (Ensembl Database 2016.12.01) were quantified using the Transcripts Per Million method.^85^^,^^86^ Data analysis was performed using Python3 [version 3.8]^76^ and R [version 4.2.2].^77^ We used pandas [version 1.2.4],^78^ matplotlib [version 3.2.1],^79^ and gplots [version 3.1.3]^80^ to create heatmaps. Additionally, we used pandas [version 1.2.4], numpy [version 1.18.5],^81^ and sklearn [version 0.23.1]^82^ to perform the principal component analysis. We used org.Hs.e.g.,.db [version 3.16.0],^83^ DOSE [version 3.24.2],^87^ and ClusterProfiler [version 4.2.2]^88^ to perform GSEA. For gene ontology enrichment analysis, gene ontology terms were extracted from the list of genes whose expression differed by more than 2-fold.

#### Imaging mass spectrometry

A MALDI-quadrupole ion trap time-of-flight mass spectrometer (iMScope Trio; Shimadzu Corp, Kyoto, Japan) was used for data acquisition. Herein, the frozen heart tissues were dissected, and cryosections with a thickness of 10 μm were prepared using a cryostat (CM 3050S; Leica Microsystems, Wetzlar, Germany). The sections were thaw-mounted on indium tin oxide- and MAS-coated slides (#SI0100M, Matsunami Glass, Osaka, Japan) and dried in silica gel-containing plastic tubes. The slides were then sprayed with 5 mg/mL 9-aminoacridine (Merck Millipore, Burlington, MA, USA, 818362) in 80% ethanol for negative ion detection or 30 mg/mL α-cyano-4-hydroxycinnamic acid (Sigma-Aldrich, St. Louis, MO, USA, 476870) in 50% acetonitrile for positive ion detection. To visualize amino acids, we applied an on-tissue derivatization method using *p*-N,N,N,-trimethylammonioanilyl N′-hydroxysuccinimidyl carbamate iodide (TAHS) to tissue sections.^87^^,^^88^ Mass images were acquired in positive ion mode (*m/z* 100–450, *m/z* 250–630 for amino acid) and negative ion mode (*m/z* 85–400, 300-670) with 80 laser shots per spot. Mass spectra acquired from each measurement point in a 25-μm pitch were reconstructed as 2D heatmaps using IMAGEREVEAL MS software (Shimadzu Corp, Kyoto, Japan). After raster scanning for IMS, the sections were washed with acetone and stained with hematoxylin and eosin (HE) according to the manufacturer’s protocol (Muto Pure Chemicals, Tokyo, Japan). The HE-stained slides were scanned using a virtual slide scanner (Nanozoomer-XR, Hamamatsu Photonics, Hamamatsu, Japan). The host myocardium, hiPSC-CM and hiPSC-T grafts were identified in each image.

#### PET studies

Before [^18^F]FDG administration, the mice were fasted from the previous evening. In the all PET experiments, the NOG mice were anesthetized with 5% (v/v) isoflurane before PET scanning, and a custom-made catheter with a needle was inserted into the tail vein for injection. Subsequently, the mice maintained under anesthesia with 1.5% (v/v) isoflurane were secured in a custom-designed chamber and placed in a PET scanner (Inveon, Siemens Medical Solutions, Knoxville, TN, USA). The body temperature of the mice was maintained with a heated water (38°C–40°C) circulation system (T/Pump TP401, Gaymar Industries, Orchard Park, NY, USA). Static emission scans (10 min) were performed 20 min after a bolus injection of [^18^F]FDG (7–10 MBq/head) and [^11^C]Met (35–40 MBq/head). On the other hand, dynamic emission scans were performed in a 3D list mode for 60 min (1 min × 4 frames, 2 min × 8 frames, and 5 min × 8 frames) after a bolus injection of [^18^F]fPhe (13–15 MBq/head). To determine the anatomical localization, the chamber with the mice was moved to a small-animal computed tomography (CT) system (CosmoScan GX, Rigaku, Tokyo, Japan) under anesthesia with 2%–3% (v/v) isoflurane after the PET scan. CT images were acquired on non-enhanced scans for 16 s using the X-ray source set at 70 kVp and 114 μA. To acquire the tissue time-activity curves of [^18^F]fPhe, dynamic PET images and average CT attenuation images were combined using PMOD software (version 3.4, PMOD Technologies, Zurich, Switzerland), and the volumes of interest were manually drawn on the heart, skeletal muscle of the leg, and intensive region of the hiPSC-Ts. The standard uptake value (SUV) was calculated as follows: SUV = (radioactivity per milliliter tissue/injected radioactivity) × gram body weight.

#### *Ex vivo* autoradiography

After PET and CT imaging, all mice were deeply anesthetized with 5% (v/v) isoflurane and transcardially perfused with saline. The hearts and subcutaneous hiPSC-Ts were quickly removed, embedded in the OCT compound, and frozen in dry ice powder. The frozen hearts and subcutaneous hiPSC-Ts sections (20-μm-thick) were prepared using a microtome (NX70; Thermo Scientific, MA, USA) and thaw-mounted on glass slides (Matsunami Glass, Tokyo, Japan). The sections were exposed to an imaging plate (BAS-MS2025; FUJIFILM, Tokyo, Japan) for 15 min (for [^18^F]FDG), 20 min (for [^18^F]fPhe) or 1 h (for [^11^C]Met). Radioactivity in the heart and subcutaneous hiPSC-Ts was detected by scanning the imaging plate using the BAS-50000 system (FUJIFILM, Tokyo, Japan).

### Quantification and statistical analysis

Statistical analysis was conducted using GraphPad Prism 9 software. Sarcomere length of hiPSC-CMs in images of immunohistochemistry was analyzed by Tukey’s multiple comparisons test (*n* = 20 at each time point). Data of graft-to-host ratio in IMS were analyzed by Dunn’s multiple comparisons test (*n* = 5 at each week). The mean values of the radioactivity of [^18^F]fPhe in hiPSC-Ts and skeletal muscles were analyzed by Wilcoxon Signed Rank Test (*n* = 6).

## Supplemental information

Document S1. Figures S1–S4

Table S1. Metabolites detected in IMS experiments and their signal intensity ratio of hiPSC-CM grafts to host myocardium, related to Figure 2

Table S2. Metabolites detected in IMS experiments and their signal intensity ratio of hiPSC-T grafts to host myocardium, related to Figure 4
