# Supplementary material for: Metabolic changes of human induced pluripotent stem cell-derived cardiomyocytes and teratomas after transplantation
Source: iScience. 2024 Oct 23;27(11):111234. doi: 10.1016/j.isci.2024.111234 (PMC11576393; doi:10.1016/j.isci.2024.111234)
Supplement: Document S1. Figures S1–S4 [file mmc1.pdf]

iScience, Volume *27*

Supplemental information

Metabolic changes of human induced pluripotent stem cell-derived cardiomyocytes and teratomas after transplantation

Yusuke Soma, Shugo Tohyama, Akiko Kubo, Tomoteru Yamasaki, Noriko Kabasawa, Kotaro Haga, Hidenori Tani, Yuika Morita-Umei, Tomohiko C. Umei, Otoya Sekine, Masashi Nakamura, Taijun Moriwaki, Sho Tanosaki, Shota Someya, Yujiro Kawai, Masatoshi Ohno, Yoshikazu Kishino, Hideaki Kanazawa, Jun Fujita, Ming-Rong Zhang, Makoto Suematsu, Keiichi Fukuda, and Masaki Ieda

Supplemental Information

Metabolic changes of human induced pluripotent stem cell-derived cardiomyocytes and teratomas after transplantation

Yusuke Soma, Shugo Tohyama, Akiko Kubo, Tomoteru Yamasaki, Noriko Kabasawa, Hidenori Tani, Yuika Morita-Umei, Tomohiko C Umei, Otoya Sekine, Masashi Nakamura, Kotaro Haga, Taijun Moriwaki, Sho Tanosaki, Shota Someya, Yujiro Kawai, Masatoshi Ohno, Yoshikazu Kishino, Hideaki Kanazawa, Jun Fujita, Ming-Rong Zhang, Makoto Suematsu, Keiichi Fukuda and Masaki Ieda

Figure S1

A

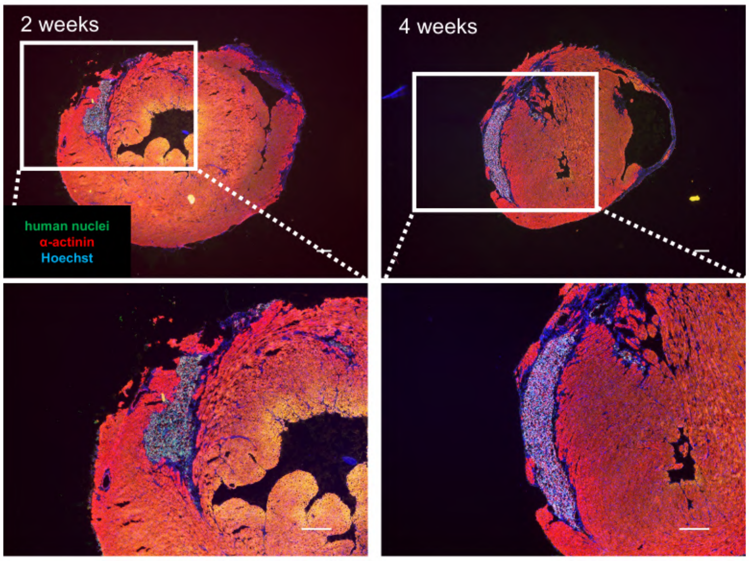

B

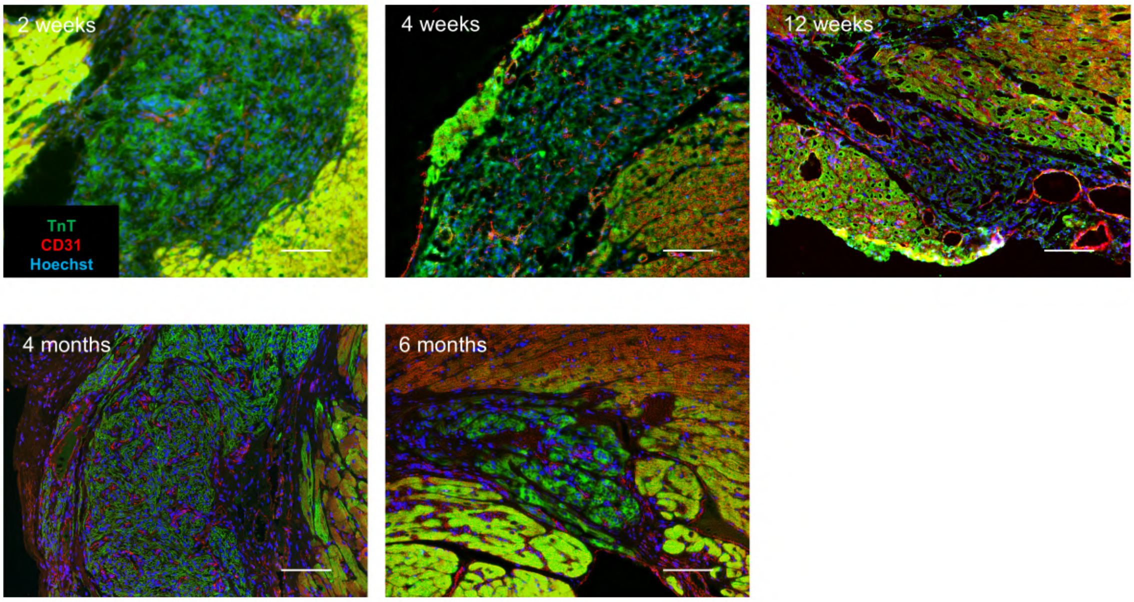

C

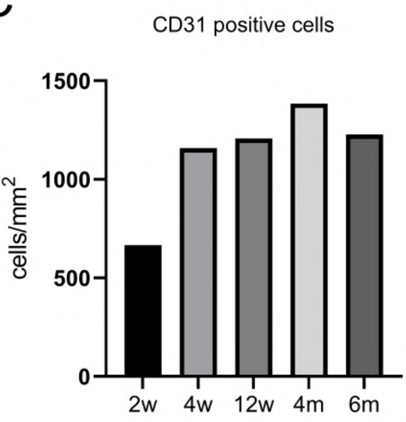

Figure S1. Identification of hiPSC-CM grafts and evaluation of their vascularization

A. Identification of hiPSC-CM grafts by staining human nuclei (green) and α-actinin (red) 2 weeks and 4 weeks after transplantation. Scale bar 300 μm. **B**. Graft staining for cardiac troponin T (green) and CD31 (red). Scale bar 100μm. **C**. The number of CD31-positive cells per mm^2.

Figure S2

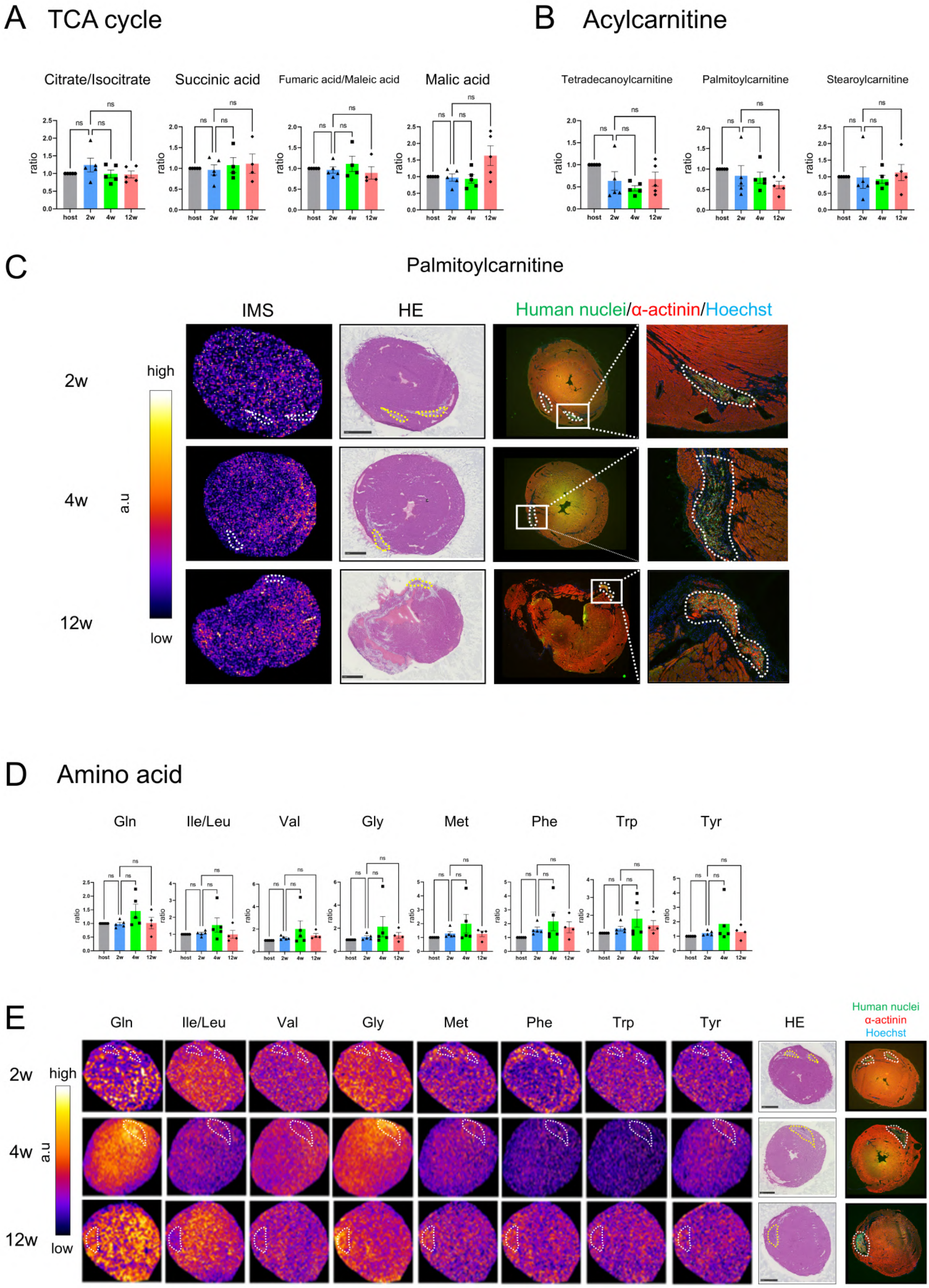
A
TCA cycle
Citrate/Isocitrate
Succinic acid
Fumaric acid/Maleic acid
Malic acid
B
Acylcarnitine
Tetradecanoylcarnitine
Palmitoylcarnitine
Stearoylcarnitine
ratio
host
2w
4w
12w
ns
C
Palmitoylcarnitine
IMS
HE
Human nuclei/α-actinin/Hoechst
2w
4w
12w
high
low
a.u
D
Amino acid
Gln
Ile/Leu
Val
Gly
Met
Phe
Trp
Tyr
E
Gln
Ile/Leu
Val
Gly
Met
Phe
Trp
Tyr
HE
Human nuclei
α-actinin
Hoechst
2w
4w
12w
high
low
a.u

Figure S2. Metabolic evaluation of hiPSC-CM grafts via IMS

A. The ratio of metabolites of the TCA cycle between hiPSC-CM grafts and host myocardium at 2, 4, and 12 weeks after transplantation (n=5, respectively; however, as for succinic acid and fumaric acid, n=4 at 4 and 12 weeks. Dunn's multiple comparisons test. Error bars represent SEM). **B**. The ratio of acylcarnitines between hiPSC-CM grafts and host myocardium at 2, 4, and 12 weeks after transplantation (n=5, respectively. Dunn's multiple comparisons test. Error bars represent SEM). **C**. Representative data showing the accumulation of palmitoylcarnitine in hiPSC-CM grafts and host myocardium measured 2, 4, and 12 weeks after transplantation. Scale bar, 1mm. **D**. The ratio of amino acids between hiPSC-CM grafts and host myocardium at 2, 4, and 12 weeks after transplantation (n=5 at 2 and 4 weeks, n=4 at 12 weeks. Dunn's multiple comparisons test. Error bars represent SEM). **E**. Representative data showing the accumulation of amino acids in hiPSC-CM grafts and host myocardium measured 2, 4, and 12 weeks after transplantation. Scale bar, 1mm.

Figure S3

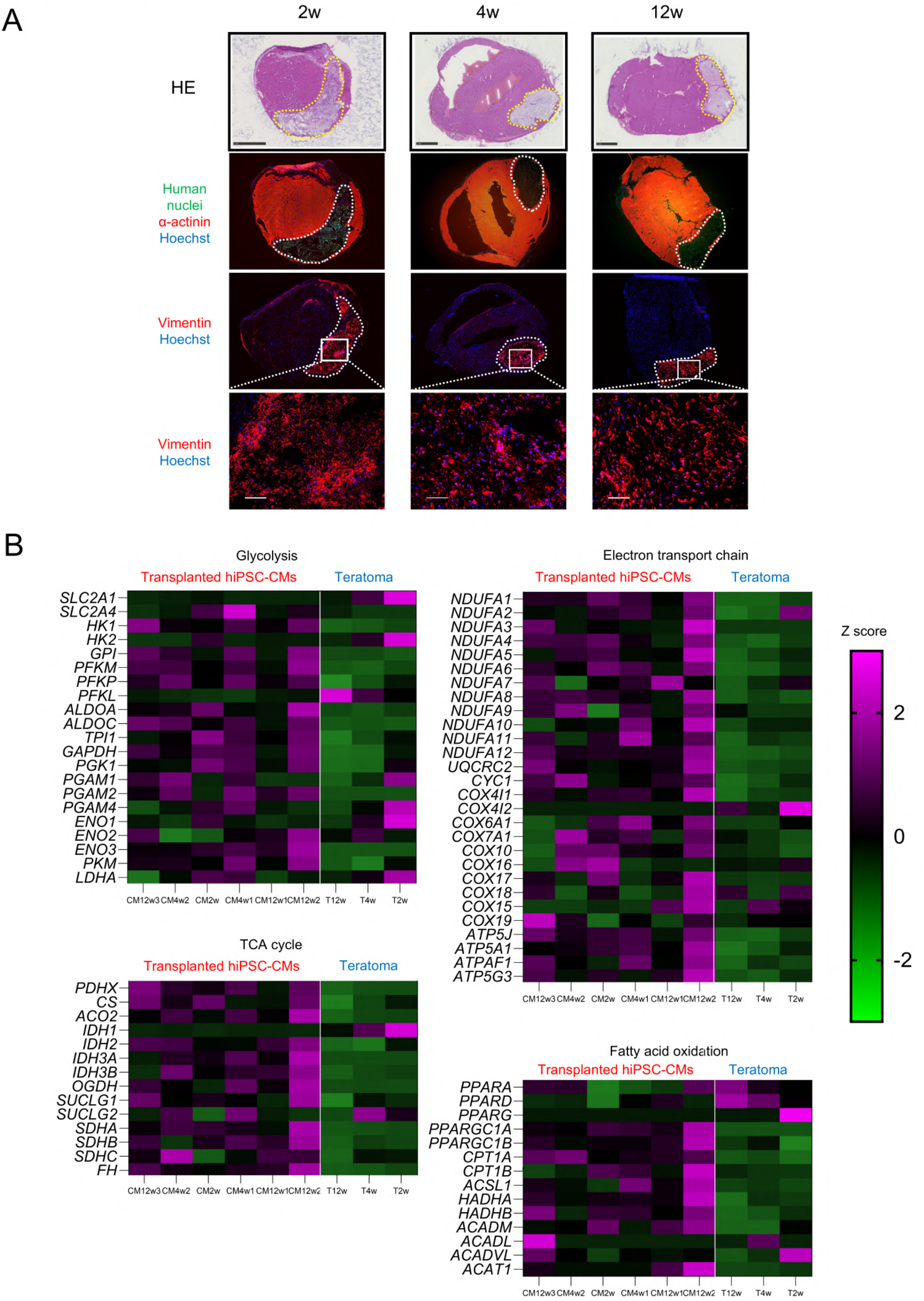
A
2w
4w
12w
HE
Human nuclei
α-actinin
Hoechst
Vimentin
Hoechst
Vimentin
Hoechst
B
Glycolysis
Transplanted hiPSC-CMs
Teratoma
SLC2A1
SLC2A4
HK1
HK2
GPI
PFKM
PFKP
PFKL
ALDOA
ALDOC
TPI1
GAPDH
PGK1
PGAM1
PGAM2
PGAM4
ENO1
ENO2
ENO3
PKM
LDHA
CM12w3 CM4w2 CM2w CM4w1 CM12w1 CM12w2 T12w T4w T2w
TCA cycle
Transplanted hiPSC-CMs
Teratoma
PDHX
CS
ACO2
IDH1
IDH2
IDH3A
IDH3B
OGDH
SUCLG1
SUCLG2
SDHA
SDHB
SDHC
FH
CM12w3 CM4w2 CM2w CM4w1 CM12w1 CM12w2 T12w T4w T2w
Electron transport chain
Transplanted hiPSC-CMs
Teratoma
NDUFA1
NDUFA2
NDUFA3
NDUFA4
NDUFA5
NDUFA6
NDUFA7
NDUFA8
NDUFA9
NDUFA10
NDUFA11
NDUFA12
UQCRC2
CYC1
COX4I1
COX4I2
COX6A1
COX7A1
COX10
COX16
COX17
COX18
COX15
COX19
ATP5J
ATP5A1
ATPAF1
ATP5G3
CM12w3 CM4w2 CM2w CM4w1 CM12w1 CM12w2 T12w T4w T2w
Z score
2
0
-2
Fatty acid oxidation
Transplanted hiPSC-CMs
Teratoma
PPARA
PPARD
PPARG
PPARGC1A
PPARGC1B
CPT1A
CPT1B
ACSL1
HADHA
HADHB
ACADM
ACADL
ACADVL
ACAT1
CM12w3 CM4w2 CM2w CM4w1 CM12w1 CM12w2 T12w T4w T2w

Figure S3. Metabolic differences between hiPSC-CM and -T grafts revealed using RNA sequencing

A. Images of hiPSC-Ts. (Top) Hematoxylin and eosin staining. Scale bar, 1 mm. (Second) Immunostaining for human nuclei (green) and α-actinin (red). (Third) Immunostaining for vimentin (red) at low magnificent. (Bottom) Immunostaining for vimentin (red) at high magnificent. Scale bar, 100 μm. B. Heatmaps of characteristic gene expression related to glycolysis, TCA cycle, ETC, and fatty acid oxidation, contrasting hiPSC-CM and -T grafts.

Figure S4

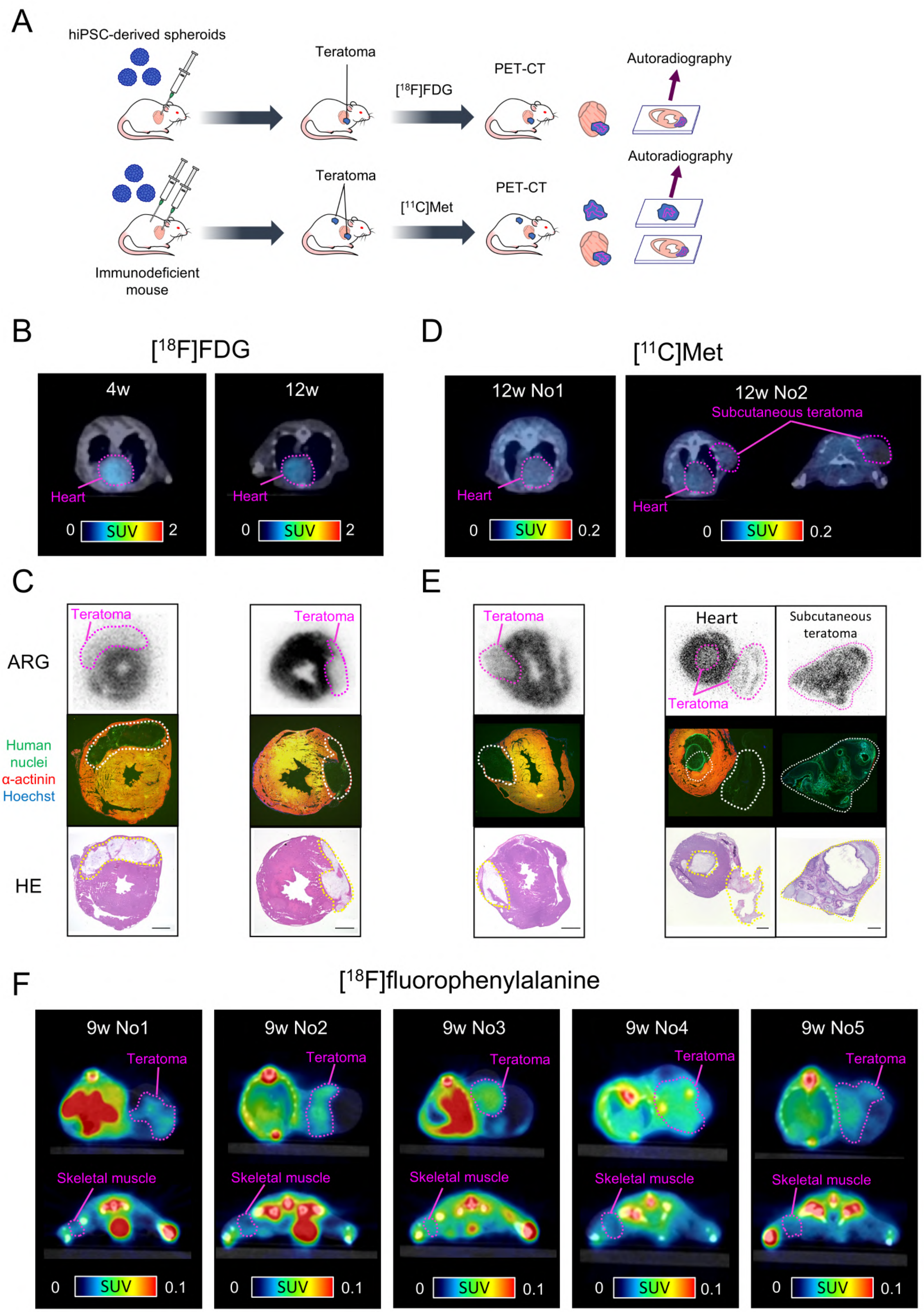

A
hiPSC-derived spheroids
Teratoma
[18F]FDG
PET-CT
Autoradiography
Teratoma
[11C]Met
PET-CT
Autoradiography
Immunodeficient mouse
B
[18F]FDG
4w
12w
Heart
Heart
0 SUV 2
0 SUV 2
D
[11C]Met
12w No1
12w No2
Subcutaneous teratoma
Heart
Heart
0 SUV 0.2
0 SUV 0.2
C
Teratoma
Teratoma
ARG
Human nuclei
α-actinin
Hoechst
HE
E
Teratoma
Heart
Subcutaneous teratoma
Teratoma
F
[18F]fluorophenylalanine
9w No1
9w No2
9w No3
9w No4
9w No5
Teratoma
Skeletal muscle
0 SUV 0.1

Figure S4. PET images of hiPSC-Ts after administration of [^{18}F]FDG, [^{11}C]Met, and [^{18}F]fPhe

A. Scheme of the PET imaging experiment using [^{18}F]FDG and [^{11}C]Met. **B.** Representative two PET images of intracardial hiPSC-Ts by [^{18}F]FDG 4 and 12 weeks after transplantation. **C**. Autoradiography of hiPSC-Ts after PET imaging by [^{18}F]FDG. **D.** PET images of intracardial and subcutaneous hiPSC-Ts by [^{11}C]Met 12 weeks after transplantation (n=2). **E**. Autoradiography of hiPSC-Ts after PET imaging by [^{11}C]Met. **F.** PET images of subcutaneous hiPSC-Ts by [^{18}F]fPhe 9 weeks after transplantation. PET images of five mice are presented, excluding the image of one mouse which are shown in Figure 4E.
